# Defining COMMD4 as an anti-cancer therapeutic target and prognostic factor in non-small cell lung cancer

**DOI:** 10.1038/s41416-020-0899-2

**Published:** 2020-05-22

**Authors:** Amila Suraweera, Alex Duff, Mark N. Adams, Christian Jekimovs, Pascal H. G. Duijf, Cheng Liu, Matthew McTaggart, Sam Beard, Kenneth J. O’Byrne, Derek J. Richard

**Affiliations:** 1 Queensland University of Technology (QUT), School of Biomedical Sciences, Institute of Health and Biomedical Innovation and Translational Research Institute, 37 Kent Street, Woolloongabba, QLD 4102 Australia; 2 Princess Alexandra Hospital, 199 Ipswich Road, Woolloongabba, QLD 4102 Australia; 3 University of Queensland Diamantina Insitute, Translational Research Institute, 37 Kent Street, Woolloogabba, QLD 4102 Australia; 4 Envoi Specialist Pathologists, Brisbane, QLD Australia; 5 Faculty of Medicine, University of Queensland, Herston, QLD 4006 Australia; 6 The Conjoint Gastroenterology Laboratory, QIMR Berghofer Medical Research Institute, Herston, QLD 4006 Australia

**Keywords:** Non-small-cell lung cancer, Non-small-cell lung cancer

## Abstract

**Background:**

Non-small cell lung cancers (NSCLC) account for 85–90% of all lung cancers. As drug resistance critically impairs chemotherapy effectiveness, there is great need to identify new therapeutic targets. The aims of this study were to investigate the prognostic and therapeutic potential of the copper-metabolism-domain-protein, COMMD4, in NSCLC.

**Methods:**

The expression of COMMD4 in NSCLC was investigated using bioinformatic analysis, immunoblotting of immortalised human bronchial epithelial (HBEC) and NSCLC cell lines, qRT-PCR and immunohistochemistry of tissue microarrays. COMMD4 function was additionally investigated in HBEC and NSCLC cells depleted of COMMD4, using small interfering RNA sequences.

**Results:**

Bioinformatic analysis and in vitro analysis of *COMMD4* transcripts showed that *COMMD4* levels were upregulated in NSCLC and elevated *COMMD4* was associated with poor prognosis in adenocarcinoma (ADC). Immunoblotting demonstrated that COMMD4 expression was upregulated in NSCLC cells and siRNA-depletion of COMMD4, decreased cell proliferation and reduced cell viability. Cell death was further enhanced after exposure to DNA damaging agents. COMMD4 depletion caused NSCLC cells to undergo mitotic catastrophe and apoptosis.

**Conclusions:**

Our data indicate that COMMD4 may function as a prognostic factor in ADC NSCLC. Additionally, COMMD4 is a potential therapeutic target for NSCLC, as its depletion induces cancer cell death.

## Background

Lung cancer is the most commonly diagnosed cancer worldwide and additionally the most common cause of death from cancer.^1–3^ In 2018, there were ~2.1 million new lung cancer cases diagnosed and 1.8 million deaths worldwide resulting from lung cancer, accounting for 11.6% of the total cancer burden.^4^

Non-small cell lung cancers (NSCLC) account for 85–90% of lung cancers and can be further classified into adenocarcinoma (ADC), squamous cell carcinoma (SCC) and large cell carcinomas (LCC) which account for ~40%, 30% and 10%, respectively, of all lung cancers.^5,6^

At present, surgery is the best option for patients with stage I–II NSCLC, with the 5-year survival rate reaching 80–90% for stage IA and 73%, 65% and 56% for stage IB, IIA and IIB, respectively.^7^ Nevertheless, most NSCLC cases present at advanced stage IIIB or IV, which is incurable, and in some patients, relapse after surgery is observed. Although platinum-based chemotherapy remains commonly used for the majority of NSCLC patients,^8^ acquired resistance and relapse pose a significant challenge in the long-term treatment of NSCLC.^9^ For early-stage NSCLC patients who are not suitable for surgery, radiotherapy remains an important frontline treatment option^10^ and at present, there are several promising clinical trials combining radiotherapy with immune checkpoint inhibitors.^11^ Despite advances in immunotherapy and targeted therapies, the global 5-year survival rate for NSCLC remains low at 4–17%, depending on the stage and regional differences.^3,7,12^ Thus, the identification of novel therapeutic targets and cancer therapies are needed to improve patient outcome and the quality of life of NSCLC sufferers.

A family of copper metabolism gene MURR1 domain (COMMD) proteins, have shown promise as potential therapeutic targets in several cancers.^13,14^ COMMD proteins are a family of ten evolutionarily conserved proteins, characterised by the presence of a highly conserved carboxy-terminal COMM domain. COMMD proteins regulate many biological processes including copper homoeostasis, activity of the NF-κB transcription factor, cell proliferation and protein trafficking.^15,16^ Interestingly, NF-κB has been implicated in the survival of cancer cells and activated NF-κB has been implicated in cisplatin resistance.^17^ COMMD1 expression has been shown to be downregulated in several cancers, with lower expression associated with a more invasive tumour phenotype. COMMD1 inhibits tumour invasion by disrupting the dimerisation of the hypoxia-inducible factor, HIF-1α/β, a transcription factor that regulates oxygen homoeostasis.^13^ Interestingly, CIGB-552, an anti-tumour peptide, was shown to increase COMMD1 expression levels in cells, induce apoptosis and inhibit the growth of human lung cancer cells.^14^ Knockdown of COMMD7 was shown to suppress cell proliferation, migration and invasion and led to apoptosis in hepatocellular carcinoma stem cell models, suggesting COMMD7 as therapeutic target in hepatocellular carcinoma,^18^ while COMMD10 was shown to possibly function as a tumour suppressor in clear cell renal carcinoma.^19^ Interestingly, COMMD9 expression was shown to be upregulated in NSCLC cells and tissues and siRNA-mediated silencing of COMMD9 resulted in the inhibition of cell migration and proliferation, arrested cells at the G1/S phase of the cell cycle and induced autophagy of the NSCLC cells. The authors additionally demonstrated that COMMD9 attenuated p53 signalling and through its interaction with TFDP1, COMMD9 promoted TFDP1/E2F1 activation in NSCLC.^20^

Here, we have investigated the role of COMMD4 in NSCLC. COMMD4 has been shown to control NF-κB activity^21^ and regulate the activity of cullin-RING E3 ubiquitin ligases.^22^ We demonstrate that COMMD4 expression is upregulated in NSCLC and high *COMMD4* expression is prognostic for ADC patient outcome. We further show that siRNA-mediated depletion of COMMD4 markedly reduces cell proliferation and viability after exposure to double-strand DNA breaks induced by ionising radiation and camptothecin. COMMD4 depletion ultimately leads to apoptosis induced by mitotic catastrophe in NSCLC cells, suggesting COMMD4 as a promising therapeutic target in NSCLC.

## Methods

### Antibodies

The following primary antibodies were used; anti-COMMD4 (Abcam, ab115169), anti-COMMD4 (Abcam, ab219115), CK7 (Abcam, ab9021), anti-β-actin (BD Biosciences, 612656), PARP1 (Cell Signaling, 9532), α-tubulin (Sigma-Aldrich, T5168) and lamin A/C (Cell Signaling, 4777). The following secondary antibodies were used for immunoblotting; IRDye® 800CW Donkey anti-mouse, IRDye® 800CW Donkey anti-goat and IRDye® 680CW Donkey anti-rabbit (LI-COR, Inc.). The following secondary antibodies were used for immunofluorescence; Alexa Fluor® 488 donkey anti-rabbit (Life Technologies, A21206), Alexa Fluor® 488 donkey anti-mouse (Life Technologies, A21202), Alexa Fluor® 594 donkey anti-mouse (Life Technologies, A21203) and Alexa Fluor® 594 donkey anti-rabbit (Life Technologies, A21207).

### Cell culture, cell treatments and reagents

Human bronchial epithelial cells (HBEC3-KT) were maintained in keratinocyte serum-free medium supplemented with epidermal growth factor, bovine pituitary extract (Life Technologies) and 10% foetal bovine serum (FBS).^23^ A549, H1299, HCC827, H1975, H460, SKEMES-1, EBC-1, HTB-182, CRL-5889 and H226 cells were cultured in Roswell Park Memorial Institute (RPMI) 1640 medium (Life Technologies) containing 10% FBS. All cells were cultured in a humidified incubator at 37 °C/5% CO_2_ atmosphere. The histologic features and origin of all cell lines are listed in Supplementary Table 1. The HBEC3-KT cell line was a gift from Professor John D Minna, University of Texas Southwestern Medical Centre, USA.^23^ All NSCLC cell lines were validated by the Genome Research Centre, Queensland University of Technology, Australia.

Camptothecin was purchased from Sigma-Aldrich and irradiations were performed at room temperature using a ^137^Cs source (Gammacell 40 Exactor [MDS Nordion]; dose rate 1.1 Gy/min).

Hoechst 33342 was purchased from ThermoFisher Scientific and Phalloidin-Atto 488 from Sigma-Aldrich.

### Small interfering RNA (siRNA) and transfections

Cells were transfected with COMMD4 siRNA #2 (CCAUGUCCCUCUCAGCAGA[dT][dT] MISSION® siRNA, Sigma-Aldrich) or COMMD4 siRNA #3 (GUCUGCAGCCUACGCAUGA[dT][[dT] MISSION® siRNA, Sigma-Aldrich) to deplete COMMD4 levels in cells. Alternatively, a scrambled siRNA, control siRNA (MISSION® siRNA Universal Negative Control #1, Sigma-Aldrich) was used. siRNA was transfected into cells using RNAiMax (Life Technologies) according to the manufacturer’s instructions. Samples were analysed 48–72 h post-transfection. HBEC3-KT or NSCLC cells were transfected with a COMMD4 siRNA-resistant plasmid, resistant to COMMD4 siRNA #2, cloned into the expression vector pCMV6-AC-3DDK containing a C-terminal FLAG tag (COMMD4-FLAG) (Sigma-Aldrich). Transfections were performed using FuGENE® HD (Promega) according to the manufacturer’s instructions and samples were analysed 24 h post-transfection.

### Collection of lysates and immunoblotting analyses

Whole-cell lysates were washed with phosphate-buffered saline and lysed in ice-cold NP40 buffer (20 mM HEPES pH 8, 150 mM KCl, 10 mM MgCl_2_, 0.5 mM EDTA, 0.2 % NP40, 0.5 mM DTT, 5% glycerol, 1X protease inhibitor cocktail, 1X phosphatase inhibitor cocktail (Roche) and 1X Pierce Universal Nuclease for cell lysis (Thermo-Fisher)). 15 μg of cell lysate were separated on 4-12% Bis-Tris Plus Bolt precast gels (Life Technologies) and immunoblotted with the indicated antibodies as previously described.^24^

### Quantitative real time PCR (qRT-PCR)

qRT-PCR was performed as previously described.^25^ *COMMD4* transcript levels (forward primer 5′-CGCAACTTCACAGAGGACAT-3′ and reverse primer 5′-TTGGCGCGATGAGGTTC-3′) were normalised to *7SL* transcript levels (forward primer 5′-ATCGGGTGTCCGCACTAAGTT-3′ and reverse primer 5′-CAGCACGGGAGTTTTGACCT-3′) using the comparative C_T_ method.

### Patient samples and immunohistochemistry, imaging and analysis

Ethics committee-approved tissue microarrays (TMA) containing squamous and adenocarcinoma tissue arrays were purchased from US Biomax Inc (LC808b and LC706a). Immunohistochemistry (IHC) was performed using the Ventana Discovery Ultra (Ventana) automated IHC stains (Roche). Deparaffinisation was performed at 50 °C for 8 min in EZ prep solution (Ventana). The temperature was increased to 60 °C and the slides were baked for a further 24 min. Cell conditioning was performed in CC1 buffer (Tris-EDTA pH 7.8) at 95 °C for 44 min, after which the slides were blocked in CM buffer at 37 °C for 4 min. For COMMD4 antibody staining, slides were incubated with the anti-COMMD4 antibody diluted 1:100 in PBS for 1 h at ambient temperature and CK7 diluted 1:1000 in PBS for 1 h at ambient temperature. The enzyme conjugate, Anti-HQ HRP was added and incubated at 37 °C for 16 min and then the corresponding secondary antibody was added and incubated at 37 °C for 16 min. Heat-induced epitope retrieval was performed in either Tris-EDTA, pH 7.8 buffer or citrate buffer, pH 6.0 for 44 min at 95 °C. Secondary antibody alone was used as the negative control.

The slides were subsequently imaged using the Vectra III Spectral Scanner (PerkinElmer). Briefly, whole slide images were taken at ×10 objective, after which the slide images were uploaded to Phenochart (version 1.0.2) (PerkinElmer) for core annotation. The core annotation on Phenochart matched the layout of the TMA. The Multi-Spectral Images (MSI) of each core on the slides were taken on the Vectra III Spectral Scanner at ×20 objective utilising the slide annotation from Phenochart.

Analysis of the MSI obtained from the Vectra III Spectral Scanner was performed on InForm Cell Analysis Software (PerkinElmer). 10 cores from ADC and SCC NSCLC Tissue Micro Array’s (TMA’s) were selected and ‘Haematoxylin and Eosin’ (H&E) were prepared using the InForm software and were sent to C.L (Consulting Pathologist) to identify cancer regions in the cores. Five of these cores were selected (the Algorithm Set) to train the algorithm in the InForm Cell Analysis Software. Filters were selected for each protein from the InForm software library, specifically Opal 570 for Rhodamine 6G, Texas Red for Red 610 and DAPI filters. The algorithm was trained for tissue segmentation based on the pathologist notated H&E cores, with ‘Red’ for tumour, ‘Green’ for Stromal/Other tissue and ‘Blue’ for negative regions. Cell Segmentation of the Algorithm Set was performed using the DAPI stain. The following values were used for the nuclear segmentation: nuclei size: minimum—50; maximum—350. Minimum signal—0.75; split—2.0; nuclei—0.40. Cytoplasmic segmentation was set at 15. The Algorithm was tested against the other 5 ‘H&E’ cores to ensure the tissue segmentation worked. All cores were then analysed using the saved Algorithm. Staining intensity was evaluated by a pathologist (C.L) in parallel and assessed on a semiquantitative scale, with scores based on staining intensity (0 = no staining, 1 = weak staining, 2 = moderate staining, 3 = strong staining).

Scores were separated by the median and correlated with the available clinicopathological parameters, including cancer type, age, sex, grade, stage and TNM Score using the Chi^2^-Test.

### Immunofluorescence microscopy and high-content microscopy

Cells transfected with control or COMMD4 siRNA were grown in optical glass bottom 96-well plates (Cellvis) and processed as previously described.^25^ Images were captured using a Delta Vision PDV microscope, ×600/1.42 or ×100/1.42 Oil objective (Applied Precision, Inc). All immunofluorescence figures were assembled using Adobe Photoshop CS6. High-content microscopy was performed using the INCell Analyzer 2200 Imaging System (GE Healthcare Life Sciences). Images were analysed using the INCell Investigator software (GE Healthcare Life Sciences) and a minimum of 150 nuclei quantified per independent experiment.

### Immunofluorescence microscopy for evaluating mitotic catastrophe

HBEC3-KT and NSCLC cells grown in 96-well plates, as above, were stained with either anti-lamin or anti-α-tubulin antibodies or with phalloidin. The presence of two or more distinct nuclear lobes within a cell, were scored as a cell that underwent mitotic catastrophe, as previously described.^26^ At least 150 cells for each experimental condition were assessed to determine the percentage of aberrant nuclei.

### Cell proliferation, apoptosis and cell cycle assays

For cell proliferation, 3 × 10^3^ cells were seeded into clear 96-well plates. Two regions per well were imaged every 2 h over a period of 96 h using the Incucyte S3 (Essen BioScience, Ann Arbor MI). The captured images were analysed using Incucyte S3 software (Essen BioScience).

Viable and apoptotic cells were measured using the Annexin V-FITC apoptosis kit using the methodology recommended by the manufacturer (Promega). HBEC3-KT and NSCLC cells were trypsinised and resuspended to 1 × 10^6^ cells/mL in binding buffer with 1:40 dilution of 488-conjugated anti-annexin antibody, followed by a 20 min incubation. Following the incubation, cells were washed with 1 X binding buffer and resuspended in 1 X binding buffer containing 1:20 dilution of propidium iodide (1 mg/mL). The cytoFLEX flow cytometer (Beckman Coulter) was used to measure the fluorescence with the software CytExpert for data acquisition. At least 20,000 events were measured per sample. Annexin FITC was used at an excitation of 488 nm, emission filter 525/40 and filter range of 505-545 nm. Propidium iodide was used at an excitation of 561 nm, dichroic filter 635LP, emission filter 585/42 and a filter range of 564-606 nm. Doublets were excluded from the analysis and forward and side-scatter profiles were gated on the dot plot. Staurosporine (Sigma-Aldrich) was used at a concentration of 5 μM for 3 h, as a positive control to induce apoptosis, as previously described.^27^ Data were analysed using the FlowJo v10 software.

To measure cell cycle distribution, cells were lifted by trypsinisation and resuspended to 1 ×10^6^ cells/mL in 70 % ethanol for 24 h at -20 °C. Following the incubation, the cells were subsequently washed with PBS and resuspended in 500 μL of PBS for 1 h at 37 °C, containing 1 mg/mL propidium iodide and RNase A (Merck) at a concentration of 0.5 mg/mL. Cell cycle analysis was subsequently performed with the cytoFLEX flow cytometer with the software CytExpert for data acquisition, using the methodology and analysis as previously described.^25,28^ At least 20,000 events were measured per sample. Propidium iodide was used at an excitation of 561 nm with a 610/20 + 001 filter. Doublet discrimination was performed and forward and side-scatter profiles, as well as PI cell cycle analysis area and width, were gated on the dot plots. Data were analysed using the FlowJo v10 software.

### Cell proliferation and apoptosis using digital holographic imaging

For proliferation and apoptosis, 3 × 10^3^ cells were seeded into lumox 96-well plates (Sarstedt). Four regions per well were imaged every 1 h over a period of 96 h using the HoloMonitorM4 (Phase Holographic Imaging). The captured images were analysed using HStudio software (Phase Holographic Imaging).

### Clonogenic cell viability assays

Clonogenic cell viability assays were carried out as previously described.^24^ Briefly, following transfection with control or COMMD4 siRNA, 400 cells for each treatment were plated in well of a 6-well plate (Corning), subsequently treated with irradiation or camptothecin the following day and allowed to recover for 8–10 days. Each condition was plated in triplicate and repeated three independent times. Data are represented as means ± SD and dose–response curves were generated using Graphpad Prism 8.

### Bioinformatics and statistical analysis

Kaplan–Meier Plotter (http://kmplot.com/analysis/index.php?p=service&cancer=lung)^29^ database was used to perform *COMMD4* survival analysis as previously described.^25^ The log-rank test and Cox proportional hazard analysis were used to determine the statistical significance of survival outcomes.

Data from the TCGA database (https://www.cancer.gov/about-nci/organization/ccg/research/structural-genomics/tcga),^30^ with samples sizes of 515, 503 and 1018 for ADC, SCC and NSCLC, respectively, were used to assess *COMMD4* transcript expression levels across NSCLC stages and histologies compared to adjacent healthy tissue. Box plots show median expression levels with interquartile ranges and notches show the 95% confidence intervals. Significance levels were determined by unpaired Mann–Whitney *U* tests.

Data are presented as the mean plus or minus SD from at least three independent experiments. Statistical analyses were performed using a two-tailed non-paired Student’s *t*-test. Experiments containing multiple independent measurements, such as *COMMD4* mRNA and protein expression, dose–response and growth curves were compared using paired Student’s *t*-test from at least three independent experiments. The level of significance was set at *P ≤ 0.05 and **P ≤ 0.005. No statistical methods were used to predetermine sample size.

## Results

### *COMMD4* gene transcripts are upregulated in NSCLC and associated with poor patient outcome

**Fig. 1:**
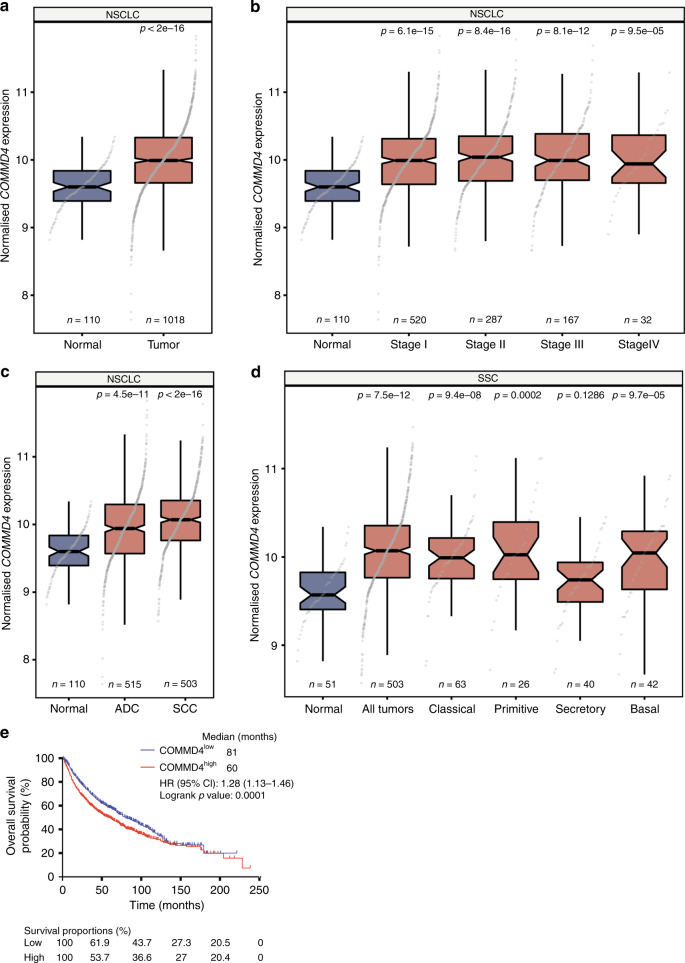
COMMD4 gene transcripts are upregulated in NSCLC and this is associated with poor patient outcome. **a**–**c** Box plots of COMMD4 transcripts comparing normal to tumour tissue (**a**), non-malignant to stages I–IV (**b**) and the comparison between the expression of COMMD4 in adenocarcinoma (ADC) and squamous cell carcinoma (SCC) (**c**). **d** A boxplot of the expression of COMMD4 in different SCC subtypes. All *p* values in **a**–**d**: Mann–Whitey *U* tests, compared to normal tissues. **e** Kaplan–Meier analysis of overall survival of 1145 NSCLC cases comparing high versus low COMMD4 expression split by median expression level. Cox proportional hazard ratio (HR) and 95% confidence interval (CI) are shown, corresponding to a *p* value of 0.0001. ADC, adenocarcinoma; SCC, squamous cell carcinoma.

In order to investigate the role of COMMD4 in NSCLC, the expression of *COMMD4* gene transcripts were first evaluated using bioinformatic analysis of The Cancer Genome Atlas (TCGA) datasets (Fig. 1a–d). *COMMD4* transcripts were analysed across NSCLC stages and histologies compared to adjacent healthy tissue. *COMMD4* expression was found to be significantly upregulated in all stages of NSCLC (*n* = 1018) compared to non-malignant tissue (*n* = 110, *p* < 2 × 10^−16^) (Fig. 1a, b). Further bioinformatic analysis revealed that *COMMD4* expression is significantly increased in both ADC and SCC subtypes, with the relative expression elevated in ADC (*n* = 515, *p* = 4.5 ×10^−11^) and SCC (*n* = 503, *p* < 2 × 10^−16^), compared to non-malignant tissue (Fig. 1c). Additionally, *COMMD4* expression is significantly higher in SCC than in ADC (*p* = 4.6 × 10^−5^) (Fig. 1c). Furthermore, *COMMD4* expression significantly increased in four of the five SCC subtypes compared to normal tissue (Fig. 1d).

While we observed an increase in *COMMD4* expression in NSCLC, we sought to determine if this increased expression within patients was linked to survival. Univariate Kaplan–Meier analysis of 1145 NSCLC cases demonstrated that NSCLC patients with high *COMMD4* expression had a poorer outcome than patients with lower *COMMD4* expression (HR = 1.28, CI: 1.13–1.46, log rank *p* = 0.0001) (Fig. 1e). To explore the prognostic value of *COMMD4* patient data, we further stratified for histological subtype. For the ADC subtype of 866 NSCLC cases, high *COMMD4* expression was found to be prognostic for patient outcome (HR = 2.01, CI: 1.57–2.56, log rank *p* = 1.1 × 10^−8^) (Supplementary Fig. 1A). On the other hand, analysis of 675 SCC cases demonstrated that *COMMD4* had no prognostic value (HR = 0.99, CI: 0.78–1.25, log rank *p* = 0.91) (Supplementary Fig. 1B). This suggests that *COMMD4* expression levels steadily increase during ADC disease progression, while they are typically continuously high during the more aggressive development of SCC.

### COMMD4 protein levels are upregulated in tissue and cell lines from NSCLC patients

The expression of COMMD4 protein was evaluated by immunohistochemistry of 74 ADC and 78 SCC patient tissue microarrays (TMA’s) (US Biomax Inc). Of the 74 ADC NSCLC cases, 20 of 74 cases (27%) exhibited COMMD4 staining solely in the nucleus and 41 of 74 (55%) cases exhibited staining solely in the cytoplasm. Within the ADC NSCLC tumours expressing COMMD4 in the nucleus, 27% of cases exhibited weak (1) staining. Within the cytoplasm of the ADC NSCLC tumours, COMMD4 expression in 1% of cases exhibited moderate (2) staining and 54% of cases exhibited weak (1) staining. For the SCC NSCLC cases, 6 of 78 cases (8%) exhibited COMMD4 staining solely in the nucleus and 8 of 78 (10%) cases exhibited staining solely in the cytoplasm. Within the SCC NSCLC tumours expressing COMMD4 in the nucleus, 8% of cases exhibited weak (1) staining. 1% of cases exhibited moderate (2) and 9% of cases exhibited weak (1) COMMD4 staining within the cytoplasm of the SCC NSCLC tumours (Table 1 and Supplementary Fig. 2). The intensity scores were stratified using the median score as cut-offs and correlated with the available clinicopathological parameters. Higher nuclear and cytoplasmic COMMD4 staining were significantly associated with ADC NSCLC (nuclear *p* = 0.0032; cytoplasmic *p* = 7.5 × 10^−9^) relative to SSC NSCLC. Higher COMMD4 expression was also associated with males (nuclear *p* = 0.0005; cytoplasmic *p* = 2.71 × 10^−5^) compared with females (Table 1 and Supplementary Fig. 2). However, our TMA data demonstrated that there was no correlation between COMMD4 staining and age, tumour grade, surgical stage or TNM score.

**Table 1:** Association of COMMD4 TMA scores with clinicopathological features.

Characteristic	Total, *n*	Nuclear intensity low < = median, *n*	Nuclear intensity high > median, *n*	Nuclear intensity Chi *p*-value	Cytoplasmic intensity low < = Median, *n*	Cytoplasmic intensity high > median, *n*	Cytoplasmic intensity Chi *p*-value
*Histological type*							
ADC	74	54	20	0.0032	33	41	7.51 × 10^−9^
SCC	78	72	6		70	8	
*Age*							
Age < = 65	109	89	20	0.6825	77	32	0.3094
Age >65	43	37	6		26	17	
*Sex*							
Male	113	101	12	0.0005	88	25	2.71 × 10^−5^
Female	38	24	14		15	23	
*Tumour grade*							
Grade 1	11	10	1	0.1700	9	2	0.4978
Grade 2	58	51	7		39	19	
Grade 3	72	55	17		46	26	
*Surgical stage*							
Stage IA–IB	54	45	9	0.8147	42	12	0.1202
Stage IIA–IIB	61	49	12		38	23	
Stage IIIA–IIIB	34	29	5		21	14	
*TNM score*							
TNM - T (1–2)	119	97	22	0.5498	84	35	0.2283
TNM - T (3–4)	33	29	4		19	14	
TNM - N 0	67	55	12	0.9857	50	17	0.0880
TNM - N 1	65	54	11		38	27	
TNM - N 2	18	15	3		14	4	

*ADC* adenocarcinoma, *SCC* squamous cell carcinoma, *TNM* tumour, node, metastasis.

**Fig. 2:**
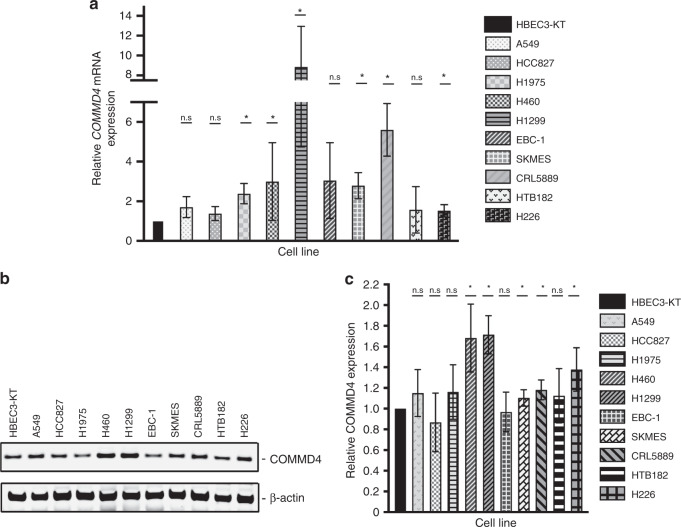
COMMD4 mRNA expression and protein expression in NSCLC cells. **a** Quantitative real-time PCR analysis of *COMMD4* transcript levels relative to the 7SL housekeeping gene compared with the immortalised epithelial cell line (HBEC3-KT) and ten NSCLC cell lines. **b** Immunoblot analysis of COMMD4 in lysates from the HBEC3-KT and ten NSCLC cells. β-actin shows the loading. **c** Quantification of the levels of COMMD4 relative to actin and then relative to HBEC-3KT (**b**). Asterix (*) denotes *p* < 0.05. n.s; not significant. Error bars represent mean ± S.D from three independent experiments.

In order to assess the in vitro function of COMMD4, the transcript and protein expression were evaluated in a panel of NSCLC cell lines and the immortalised non-tumorigenic HBEC3-KT cell line. The qRT-PCR analysis demonstrated that *COMMD4* transcripts were significantly upregulated in most of the NSCLC cell lines compared to HBEC3-KT (Fig. 2a). *COMMD4* overexpression reached statistical significance in some, but not all of the NSCLC cell lines compared with the HBEC3-KT control cell line, which could be due to the HBEC3-KT cell line adapting to the cell culture environment. Interestingly, significantly higher expression of *COMMD4* was seen in H1975 (ADC), H460 (LCC), H1299 (ADC), SKMES (SCC), CRL5889 (SCC) and H226 (SCC) cell lines compared to the HBEC3-KT cell line. Immunoblotting was subsequently performed on the same panel of cells analysed for qRT-PCR. The immunoblotting analysis corroborated our qRT-PCR, where the highest COMMD4 expression was seen in the H460 and H1299 cell lines. A549 (ADC), CRL5899 and H226 additionally showed high expression of COMMD4 (Fig. 2b, c). The cell line data did not fully match with what we observed with the bioinformatic analysis of patient samples, where we observed increased *COMMD4* expression across all ADC and SCC patients. As the HBEC3-KT cells have been immortalised with hTERT and Cdk4,^23^ their immortalisation may account for the discrepancies observed.

### COMMD4 is required for cell proliferation in NSCLC cells

**Fig. 3:**
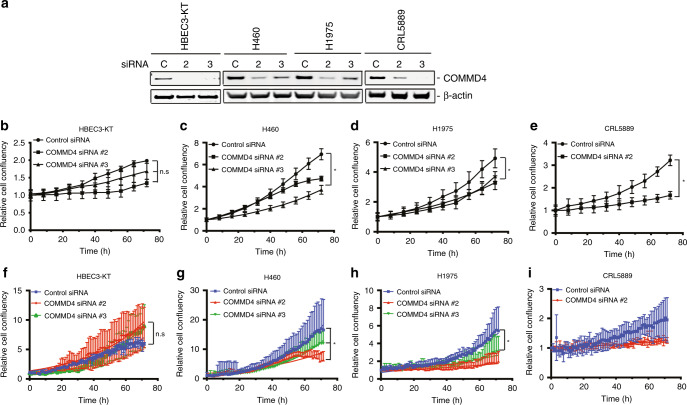
COMMD4 is required for cell proliferation in NSCLC cells. **a** Immunoblot showing the depletion of COMMD4 using control siRNA or COMMD4 siRNA #2 or #3 across HBEC3-KT and three NSCLC cell lines. β-actin shows the loading. **b**–**e** Proliferation analysis of HBEC3-KT, H460, H1975 and CRL5889 cells depleted of COMMD4 with control or siRNA #2 or #3 and analysed using the Incucyte S3 live imaging system. **f**–**i** Proliferation analysis of HBEC3-KT, H460, H1975 and CRL5889 cells depleted of COMMD4 with control or siRNA #2 or #3 and analysed using the HoloMonitor M4 live imaging system. Asterix (*) denotes *p* < 0.05. n.s, not significant. Error bars represent mean ± SD from three independent experiments.

In order to explore the function of COMMD4, we used two different siRNA sequences (#2 and #3) targeting the *COMMD4* transcript, in addition to a negative control, to deplete COMMD4 from cells (Fig. 3a). Control siRNA, COMMD4 siRNA #2 and siRNA #3 were depleted from HBEC3-KT (bronchial epithelial), H460 (LCC), H1975 (ADC) and CRL5889 (SCC) cell lines. We chose these NSCLC cell lines as they represented each NSCLC subtype. siRNA-mediated silencing of COMMD4 was assessed by immunoblotting the cell lysates. COMMD4 siRNA #2 and #3 were shown to reduce the expression of COMMD4 by ~75–85 % (Fig. 3a). Following depletion by siRNA, the Incucyte S3 was used to assess cell proliferation. We observed no significant overall changes in the growth of the HBEC3-KT cell line following COMMD4 depletion (Fig. 3b). However, in the NSCLC cell lines, there was a significant retardation of cell growth after COMMD4 depletion with siRNA #2 and #3 (Fig. 3c–e). Transfection of CRL5889 cells with COMMD4 siRNA #3 caused rapid cell death and thus, this condition was not suitable for proliferation analyses (data not shown).

We additionally analysed cell proliferation using the HoloMonitorM4 holographic imaging microscope. Similar to our observations using the Incucyte S3, we noticed substantial retardation in cell growth in the NSCLC cells representing each subtype, after depletion of COMMD4 with siRNA #2 and #3 and observed no significant reduction in cell growth in the HBEC3-KT control cell line depleted of COMMD4 (Fig. 3f–i).

### COMMD4 depletion induces mitotic catastrophe in NSCLC cells

**Fig. 4:**
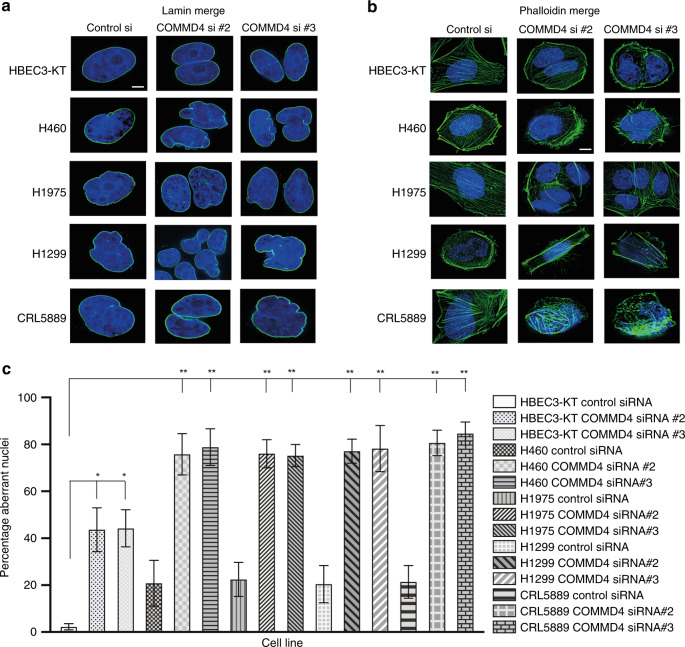
COMMD4 depletion induces mitotic catastrophe in NSCLC cells. **a**, **b** Lamin and phalloidin staining respectively in control HBEC3-KT and four NSCLC cell lines. DAPI shows the nucleus and lamin and phalloidin staining are shown in green. **c** Quantification of the percentage aberrant nuclei for (**a**) and (**b**). Asterix (*) denotes *P < 0.05 and **P < 0.005 from 150 cells counted. Error bars represent mean ± SD. Scale bar denotes 5 μm.

In order to understand the mechanism by which COMMD4 depletion led to retardation in cell growth in NSCLC cells, we investigated whether COMMD4 depletion in NSCLC cell lines resulted in cell cycle defects, using live cell imaging. We observed that cells were undergoing mitotic catastrophe. Mitotic catastrophe can be described as cell death that occurs during mitosis or as a consequence of mitotic failure,^31^ which, additionally has been recognised as an important anti-cancer strategy.^32^ We assessed three markers to determine mitotic catastrophe; lamin staining of the nuclear envelope, phalloidin staining of the actin cytoskeleton and tubulin staining of the cytoskeleton (Fig. 4a, b and Supplementary Fig. 3A and B). Mitotic catastrophe leads to morphological changes within the cell and leads to multinucleation and/or micronucleation and often results in the formation of nuclear envelopes surrounding individual clusters of missegregated chromosomes.^32,33^ While COMMD4 depletion resulted in some mitotic failure in HBEC3-KT cells, in the NSCLC cells there was significantly more induction of aberrant nuclei, micronucleation and giant multinucleated cells upon COMMD4 depletion (Fig. 4c), all morphological features associated with mitotic catastrophe.^32^

### Depleting COMMD4 sensitises NSCLC cells to irradiation and camptothecin

**Fig. 5:**
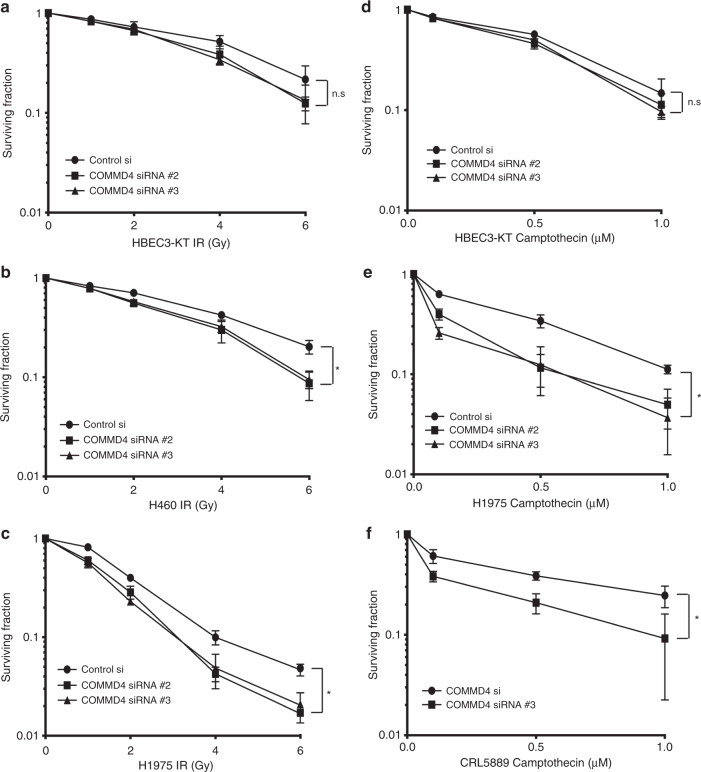
COMMD4 is required for survival following the exposure of NSCLC cells to DNA damaging agents. **a**–**c** Clonogenic cell viability assays in HBEC3-KT, H460 and H1975 NSCLC cells transfected with control or COMMD4 siRNA (#2 or #3) and treated with varying doses of irradiation (IR). **d**–**f** Clonogenic cell viability assays in HBEC3-KT, H1975 and CRL5889 cells transfected with control or COMMD4 siRNA (#2 or #3) and treated with varying doses of camptothecin as shown. Asterix (*) denotes *p* < 0.05. n.s; not significant. Error bars represent mean ± SD from three independent experiments.

Radiotherapy in early-stage NSCLC (stage I and II) has traditionally been used in patients who are medically considered inoperable, while radiotherapy remains the standard option for stage IIIA and IIIB NSCLC patients.^10,34,35^ The induction of mitotic catastrophe has previously been linked to radiation sensitivity.^26,36^ As we found that COMMD4 depletion induced mitotic catastrophe in NSCLC, we next sought to explore the therapeutic potential for depleting COMMD4 to impact radiation sensitivity. Clonogenic cell viability assays^37^ were used to determine whether COMMD4 depletion in combination with irradiation would lead to increased cell death in NSCLC compared to the control siRNA treated NSCLC cells. HBEC3-KT cells showed no significant hypersensitivity to irradiation (Fig. 5a). However, H460 and H1975 NSCLC cells depleted of COMMD4 showed significant increased sensitivity to irradiation (Fig. 5b, c). Additionally, H1299 NSCLC cells depleted of COMMD4 also showed hypersensitivity to irradiation (Supplementary Fig. 4A). These data demonstrate that COMMD4 depletion enhances the sensitivity of NSCLC cells to radiation. To confirm that the irradiation hypersensitivity was not due to off-target effects, the sensitivity of COMMD4 depletion by siRNA #2 was rescued by the transient ectopic expression of an siRNA-resistant COMMD4 overexpression construct (Supplementary Fig. 4B). Furthermore, we observed that overexpression of COMMD4 in H460 cells caused radiation resistance in these cells (Supplementary Fig. 4B). Together, these data highlight a role for COMMD4 in protecting cells from DNA double-strand breaks which are induced by irradiation.

As NSCLC cells depleted of COMMD4 were hypersensitive to irradiation, we next investigated whether COMMD4 depletion might also enhance sensitivity to camptothecin in NSCLC cells. Camptothecin class of compounds have shown promise in the treatment of many cancers including NSCLC^38^ and function by generating cytotoxic single-strand DNA breaks that are processed into DNA double-strand breaks during the S-phase of the cell cycle.^39^ While we observed no difference in the sensitivity between control and COMMD4 siRNA-depleted HBEC3-KT cells (Fig. 5d), H1975 and CRL5889 cells depleted of COMMD4 were hypersensitive to camptothecin, compared to the control siRNA transfected cells (Fig. 5e, f). This showed that COMMD4 depletion enhances camptothecin sensitivity in NSCLC cells and points to the possibility that COMMD4 holds promise as a therapeutic target. These data further confirm that COMMD4 function is necessary to protect cells from DNA double-strand breaks.

### COMMD4 depletion leads to apoptosis in NSCLC cells

**Fig. 6:**
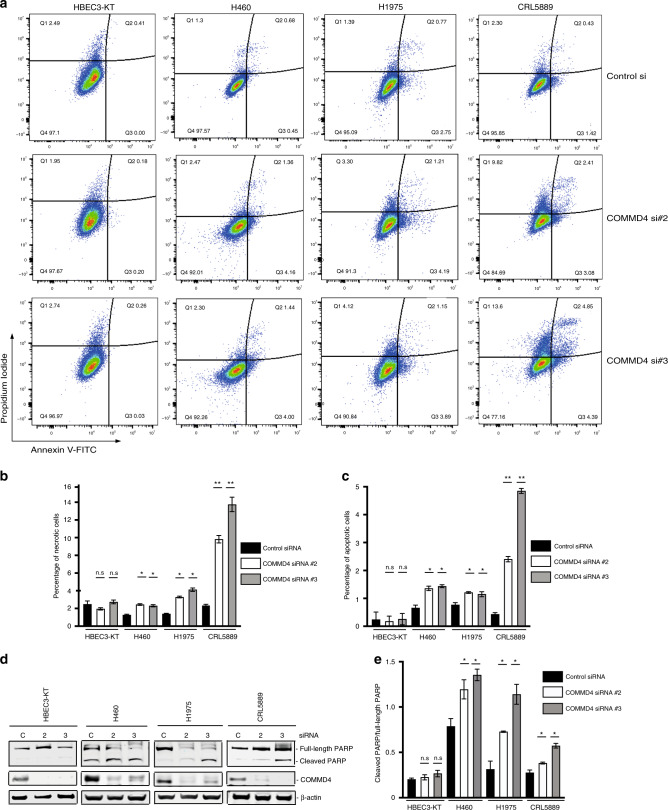
COMMD4 depletion leads to increased apoptosis in NSCLC cells. **a** Flow cytometry analyses of apoptosis with control siRNA and siRNA #2 and siRNA #3 treated HBEC3-KT, H1975 and CRL5889 cell lines. Live cells were stained with Annexin V-488 and propidium iodide and assessed using a cytoFLEX flow cytometer. The percentage of cells within each gating quadrant is listed for each panel. Live cells; lower left quadrant (Q4), apoptotic cells; right quadrants (Q2 and Q3). **b**, **c**. Quantification of (**a**). The percentage of necrotic (**b**) and apoptotic (**c**) cells is shown and was calculated from the quadrant. **d** Apoptosis measured by PARP cleavage by immunoblotting HBEC3-KT and NSCLC cell lines. **e** Quantification of (**d**). C; control siRNA, 2; COMMD4 siRNA #2, 3, COMMD4 siRNA #3. *p < 0.05, **p < 0.005, n.s; not significant. Error bars represent mean ± S.D from three independent experiments.

As we found that depleting NSCLC cells of COMMD4 led to reduced cell proliferation, reduced cell viability and induced mitotic catastrophe, we next explored whether COMMD4 depletion additionally leads to apoptosis. Apoptosis in the control HBEC3-KT and NSCLC cell lines were measured using two different cell-based assays. We initially measured apoptosis by staining for propidium iodide and Annexin V via flow cytometry. In the HBEC3-KT control and COMMD4 siRNA #2 and #3 transfected cells, we observed negligible induction of apoptosis (Fig. 6a–c). However, H460, H1975 and CRL5889 NSCLC cells depleted of COMMD4, with either siRNA #2 or #3, showed significantly increased early and late apoptosis and necrosis compared with the control siRNA transfected cells (Fig. 6a–c).

To confirm our flow cytometry data, we measured the cleavage of poly (ADP-ribose) polymerase (PARP) by immunoblotting, a hallmark of apoptosis.^40^ This demonstrated that COMMD4 depletion by siRNA resulted in PARP cleavage in NSCLC cells, indicating the induction of apoptosis. H460 cells had higher levels of PARP than the other NSCLC cells tested, however depletion of COMMD4 by siRNA resulted in elevated PARP cleavage, while we observed no cleaved PARP in the HBEC3-KT control cell line (Fig. 6d, e). These data confirm that COMMD4 depletion induces apoptosis in NSCLC cells. Importantly, the normal HBEC3-KT cell line was not impacted by COMMD4 depletion, suggesting that there may be a viable therapeutic window to exploit.

As mitotic catastrophe has been shown to precede apoptosis, necrosis and senescence,^33^ the induction of mitotic catastrophe was the likely cause of the increase in necrosis and apoptosis we previously observed when COMMD4 was depleted from the NSCLC cells.

Given that COMMD4 depletion resulted in the induction of necrosis, apoptosis and led to significantly reduced cell proliferation, we subsequently examined whether COMMD4 depletion also affected cell cycle progression in NSCLC cells. Using flow cytometry analysis, we assessed the DNA content of propidium iodide-stained asynchronous control siRNA or COMMD4-depleted, HBEC3-KT, H460, H1975 and CRL5889 cells (Supplementary Fig. 5A). Our data demonstrated no overall cell cycle changes or no significant G2/M phase cell cycle changes in the live HBEC3-KT or NSCLC cells depleted of COMMD4 that were analysed (Supplementary Fig. 5B).

## Discussion

The COMMD family of proteins presents as promising prognostic and therapeutic targets for the treatment of cancer.^15,16^ COMMD proteins contain a characteristic COMM domain within their C-terminus, which provides a platform for protein interactions.^16^ The expression of COMMD1 and COMMD9 have previously been studied in lung cancers.^14,20^ In this study, we examined the role of COMMD4 in NSCLC. NSCLC accounts for 85–90% of lung cancers and several risk factors including smoking, environmental factors and genetic susceptibility contribute towards the development of this disease.^41^

The identification of prognostic factors has the potential to have a tremendous impact on patients by selecting patient or tumour characteristics that predict a better outcome for the patient, allowing precision medicine. Prognostic markers include mRNA, miRNA, genes or proteins, however, in NSCLC, the majority of prognostic markers are proteins targeting early-stage disease.^42,43^ Meta-analyses of multiple repositories presents a cost-effective method for identifying potential prognostic factors.^43^ In this study, meta-analysis of COMMD4 expression showed that NSCLC patients expressed statistically higher levels of COMMD4 expression and this was irrespective of tumour grade or subtype. Additionally, patients with high *COMMD4* expression had a poorer outcome than patients with lower *COMMD4* expression. However, this difference was seen solely in ADC patients, where high *COMMD4* expression was found to be prognostic for patient outcome. Our bioinformatic data of patient samples was mostly consistent with our qRT-PCR analysis of NSCLC cell lines, which showed that *COMMD4* transcript was significantly upregulated in the H1975, H460, H1299, SKMES, CRL5889 and H226 NSCLC cell lines, compared to HBEC3-KT bronchial epithelial cell line. Approximately 10–40% of NSCLC patients have activating mutations in EGFR. Subsequently, these mutations lead to increased sensitivity to tyrosine kinase inhibitors.^44^ However, *COMMD4* mRNA expression did not segregate to EGFR mutation status, as there was no difference in expression between H460 and A549 cells that have wild-type EGFR and H1975 cells which contain a L858R and T790M mutation.^45^

We subsequently analysed COMMD4 protein levels from the ten NSCLC cell lines and the control HBEC3-KT cells. Immunoblotting analysis confirmed our qRT-PCR results, where our clinical and in vitro data showed elevated *COMMD4* transcript and protein levels in the majority of NSCLC analysed.

Our TMA analysis was consistent with our mRNA analysis and demonstrated that COMMD4 expression was high regardless of age, tumour grade, surgical stage or TNM score. We did, however, observe significantly higher COMMD4 protein levels in the ADC patients compared to the SCC patients. This is relevant as our mRNA expression analysis demonstrated that high levels of *COMMD4* transcript was associated with poor patient outcome. Our TMA’s did not include patient survival data and a large data set with patient outcome would be needed to confirm the prognostic potential of COMMD4 protein levels in the ADC subtype of NSCLC.

Overexpression of COMMD4 is strongly associated with NSCLC. To determine if this overexpression was essential for NSCLC cell survival, we utilised a number of NSCLC cell lines. Transfection of two independent siRNA sequences targeting COMMD4 showed significantly reduced proliferation of H460, H1975 and CRL5889 NSCLC cell lines compared to the HBEC3-KT cell line transfected with COMMD4 siRNA, suggesting that COMMD4 is required for the proliferation of these NSCLC cells.

To understand why cells depleted of COMMD4 were significantly slower at proliferating, we next looked at the phenotype of cycling COMMD4-depleted cells. Since COMMD4-depleted NSCLC cells had reduced proliferation, formed micronuclei and giant multinucleated cells, followed by decreased cell viability and apoptosis, we conclude that COMMD4 depletion in these cells induces mitotic catastrophe.^32,46^ In 2018, the International Nomenclature Committee on Cell Death defined mitotic catastrophe as a “*bona fide* oncosuppressive mechanism during the control of mitosis-incompetent cells by regulated cell death or cellular senescence”.^47^ As we also observed defects in the nuclear envelope of NSCLC depleted of COMMD4, we additionally suggest that COMMD4 depletion in these cells leads to chromothripsis, a catastrophic event frequently seen in cancer cells.^48^

If NSCLC is diagnosed at early-stage, the 5-year survival can be as high as 90% with treatment options of surgery, radiation therapy, radiofrequency ablation and chemotherapy.^7,49^ However, at the time of diagnosis, 40% patients present at advanced stage and in metastatic NSCLC, the median survival can be as low as eight months.^35,50^ As radiotherapy and chemotherapy remain a frontline treatment for NSCLC,^49^ we examined the viability of NSCLC cells depleted of COMMD4 in combination with either irradiation or the DNA damaging agent, camptothecin. Camptothecin was used to determine if any effects observed were due to DNA double-strand breaks. Clonogenic cell viability assays demonstrated that NSCLC cells depleted of COMMD4 showed hypersensitivity to irradiation and camptothecin, compared to the control siRNA transfected NSCLC cells, suggesting that COMMD4 was required for the survival of these NSCLC cells after irradiation or camptothecin treatment. This further indicates that COMMD4 may be an attractive therapeutic target in NSCLC. As the combination of COMMD4 depletion and either irradiation or camptothecin was shown to be synergistic in NSCLC, COMMD4 inhibition in combination therapy with agents that cause DNA damage may prove to be an attractive treatment option for NSCLC patients.

In addition to reduced proliferation and reduced cell viability, COMMD4 depletion resulted in the induction of apoptosis measured by Annexin V and PARP cleavage. However, we observed no overall cell cycle changes in control or NSCLC cells depleted of COMMD4.

To demonstrate on-target effects, we were able to rescue the hypersensitivity to radiation, by ectopically expressing an siRNA resistant COMMD4 expression construct in H460 cells. Interestingly, cells expressing higher levels of COMMD4 through ectopic expression showed resistance to double-strand DNA breaks generated by irradiation. This further supports our bioinformatic analyses and provides a possible explanation as to why overexpression of COMMD4 in NSCLC cells may lead to poor patient prognosis.

Copper levels within a cell have been shown to promote tumour angiogenesis, while anti-copper drugs have shown some promise as anti-cancer agents.^51^ COMMD proteins have been shown to regulate copper homoeostasis^15^ and here we demonstrate that COMMD4, in particular, may show promise as an anti-cancer target. Indeed, a novel Cu(II)-mal-picoline complex was shown to induce mitotic catastrophe and result in apoptosis in HeLa cells.^52^ Inducing mitotic catastrophe in cancer cells has proven to be a promising anti-cancer strategy.^32^ As COMMD4 transcript and protein levels are upregulated in NSCLC, targeting COMMD4 in NSCLC may provide a therapeutic window that specifically kills cancer cells.

DNA repair pathways maintain genomic stability, thereby preventing mutations that lead to diseases, such as cancer. However, genomic instability is a hallmark of all cancers^53^ and as a cancer continues to grow, genetic streamlining results in dysregulation of DNA repair pathways, followed by compensatory activities in DNA repair pathways, leading to intrinsic or acquired resistance to DNA damaging agents. Thus, targeting DNA repair proteins, halt the tumour’s compensatory repair mechanism leading to cell death and forms the basis of chemotherapeutics targeting DNA repair proteins.^54–56^

We have recently demonstrated that COMMD4 functions by regulating the monoubiquitination of H2B, following the induction of DNA double-strand breaks.^57^ We show that COMMD4 functions to limit the extent of epigenetic modification of the chromatin around the break site. Depletion of COMMD4 leads to a loss of RNF20/40 regulation and uncontrolled chromatin remodelling. As genomic instability is a hallmark of all cancers^53^ and COMMD4 transcript and protein levels are upregulated in NSCLC, targeting COMMD4 as a therapeutic target in NSCLC may lead to uncontrolled chromatin remodelling, ultimately resulting in mitotic catastrophe-induced apoptosis in these cells.

Taken together, our findings demonstrate that COMMD4 mRNA transcripts and protein are upregulated in most NSCLC patients, compared with normal tissue, implicating COMMD4 as a potential prognostic factor in the ADC subtype of NSCLC. We show that in NSCLC patient cell lines, COMMD4 plays a vital role in mediating cancer cell proliferation and the depletion of COMMD4 impairs the proliferation of these cells. Additionally, siRNA mediated depletion of COMMD4 results in necrosis, apoptosis and reduced cell viability after the induction of DNA damage, ultimately leading to the induction of mitotic catastrophe and possibly chromothripsis. These data suggest COMMD4 as a novel therapeutic target in NSCLC, acting to inhibit the proliferation of tumour cells by inducing apoptosis. These novel findings highlight the potential of a novel approach to NSCLC therapy, by targeting a novel overexpressed DNA repair protein.

## Supplementary information

Supplementary Tables and Figures

## Data Availability

All data presented in this study are included within the paper and its Supplementary files.
