# Correction: Defining COMMD4 as an anti-cancer therapeutic target and prognostic factor in non-small cell lung cancer

**DOI:** 10.1038/s41416-020-01242-4

**Published:** 2021-01-26

**Authors:** Amila Suraweera, Alex Duff, Mark N. Adams, Christian Jekimovs, Pascal H. G. Duijf, Cheng Liu, Matthew McTaggart, Sam Beard, Kenneth J. O’Byrne, Derek J. Richard

**Affiliations:** 1 Queensland University of Technology (QUT), School of Biomedical Sciences, Institute of Health and Biomedical Innovation and Translational Research Institute, 37 Kent Street, Woolloongabba, QLD 4102 Australia; 2 Princess Alexandra Hospital, 199 Ipswich Road, Woolloongabba, QLD 4102 Australia; 3 University of Queensland Diamantina Insitute, Translational Research Institute, 37 Kent Street, Woolloogabba, QLD 4102 Australia; 4 Envoi Specialist Pathologists, Brisbane, QLD Australia; 5 Faculty of Medicine, University of Queensland, Herston, QLD 4006 Australia; 6 The Conjoint Gastroenterology Laboratory, QIMR Berghofer Medical Research Institute, Herston, QLD 4006 Australia

**Correction to:** *British Journal of Cancer* (2020) **123**, 591–603; 10.1038/s41416-020-0899-2, published online 22 May 2020

Since the publication of this paper, the authors have noticed an error in the name of Kenneth J. O’Byrne, where Dr. O’Byrne’s name was incorrectly listed as Kenneth J. O’ Byrne. This has been corrected above. The original article has been corrected.

