# Supplementary material for: Defining COMMD4 as an anti-cancer therapeutic target and prognostic factor in non-small cell lung cancer
Source: Br J Cancer. 2020 May 22;123(4):591–603. doi: 10.1038/s41416-020-0899-2 (PMC7434762; doi:10.1038/s41416-020-0899-2)
Supplement: Supplementary file 1 — Supplementary Tables and Figures [file 41416_2020_899_MOESM1_ESM.pdf]

SUPPLEMENTARY TABLES AND FIGURES

Supplementary Table 1. Histology and origin of cell lines used.

Name of cell line	Histology	Origin
HBEC3-KT	Human bronchial epithelial	Established from bronchial epithelial biopsy [1]
A549	ADC	Established in 1972 from carcinomatous tissue
H1299	ADC	Established from metastatic lymph node
HCC827	ADC	Established from lung tissue
H1975	ADC	Established in 1988
H460	LCC	Established in 1982 from pleural fluid
SKMES-1	SCC	Pleural fluid of metastatic squamous cell carcinoma
EBC-1	SCC	Established from squamous cell carcinoma
HTB182	SCC	Established in 1982 from squamous cell carcinoma
CRL5889	SCC	Stage 1 squamous cell carcinoma from smoker donor tissue
H226	SCC	Established from pleural fluid

ADC; adenocarcinoma, LCC; large cell carcinoma, SCC; squamous cell carcinoma.

1. Ramirez, R.D., Sheridan, S., Girard, L., Sato, M., Kim, Y., Pollack, J. et al. Immortalization of human bronchial epithelial cells in the absence of viral oncoproteins. *Cancer Res*. **64**, 9027-9034 (2004).

SUPPLEMENTARY FIGURES

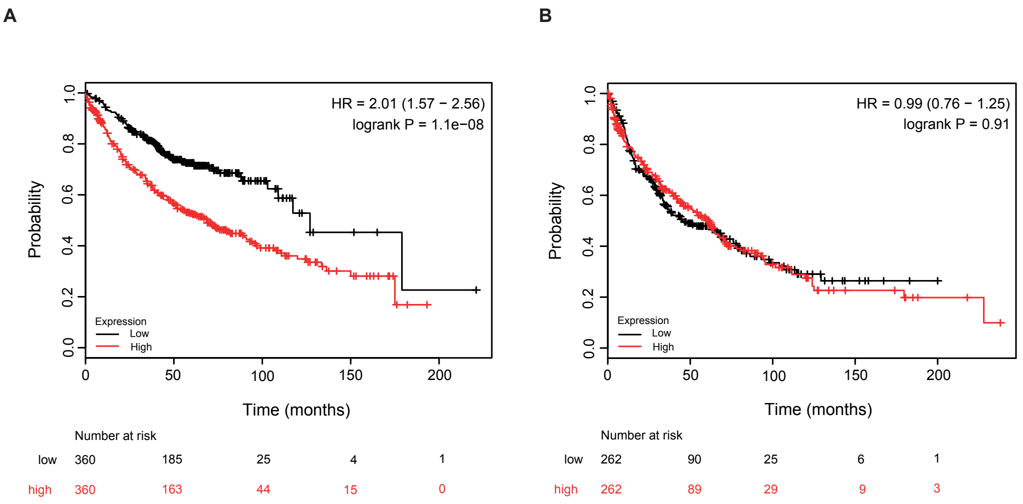

Supplementary Figure 1. Prognostic value of COMMD4 in ADC and SCC. A and B. Univariate Kaplan-Meier analysis of overall survival in ADC (A) and SCC (B) cases.HR; hazard ratio, CI; confidence interval, ADC; adenocarcinoma, SCC; squamous cell carcinoma.

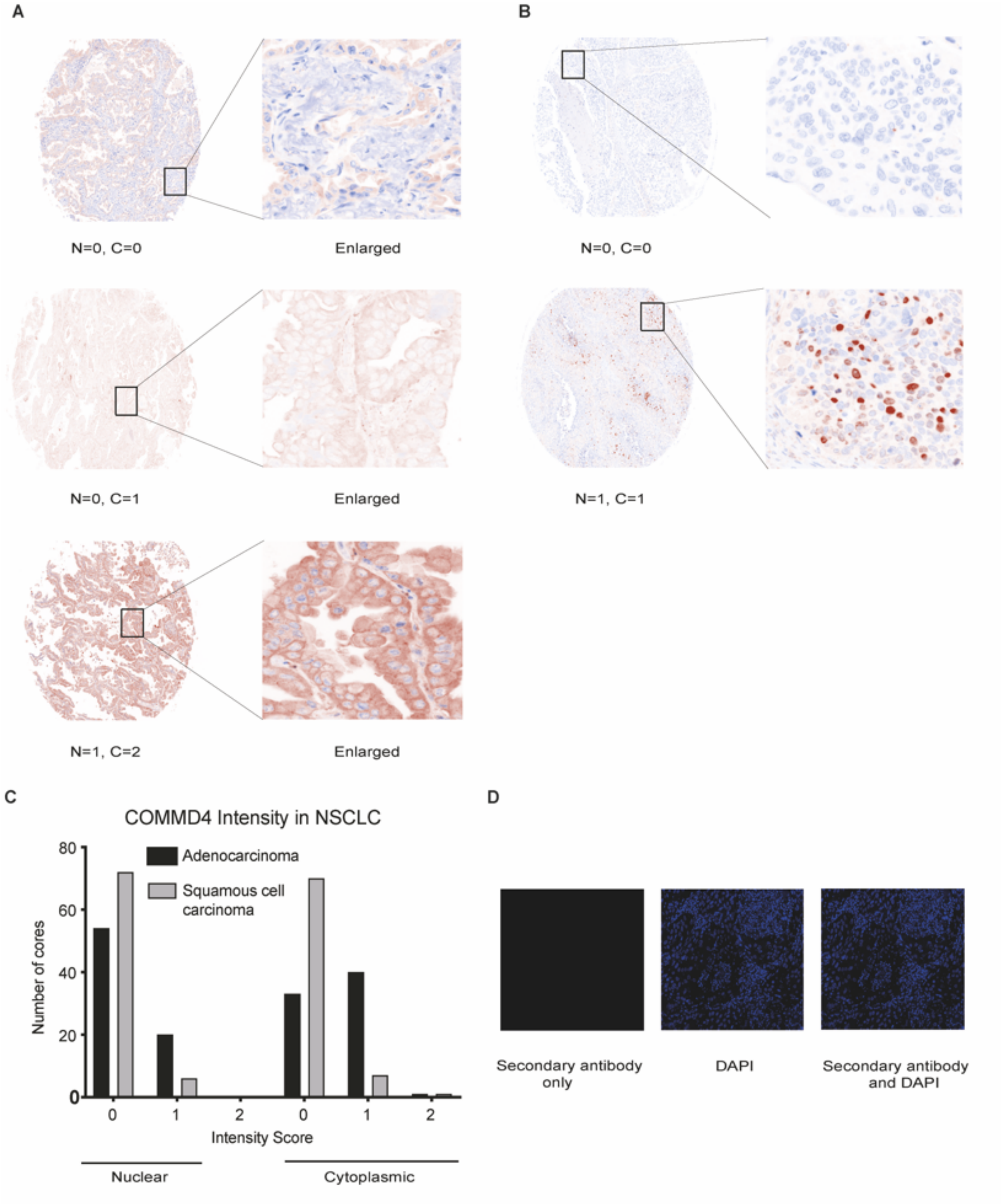

Supplementary Figure 2. COMMD4 expression. A and B. Representative images of negative (0), weak (1) and moderate (2) scores for COMMD4 nuclear and cytoplasmic staining of adenocarcinoma (A) and squamous cell carcinoma (B) TMAs. N; nuclear, C; cytoplasmic. C. Graph showing COMMD4 nuclear and cytoplasmic staining intensity of adenocarcinomas and squamous carcinomas across all TMA cores. D. Fluorescence staining of the negative control (secondary antibody only) and the DAPI nuclear staining of the TMAs.

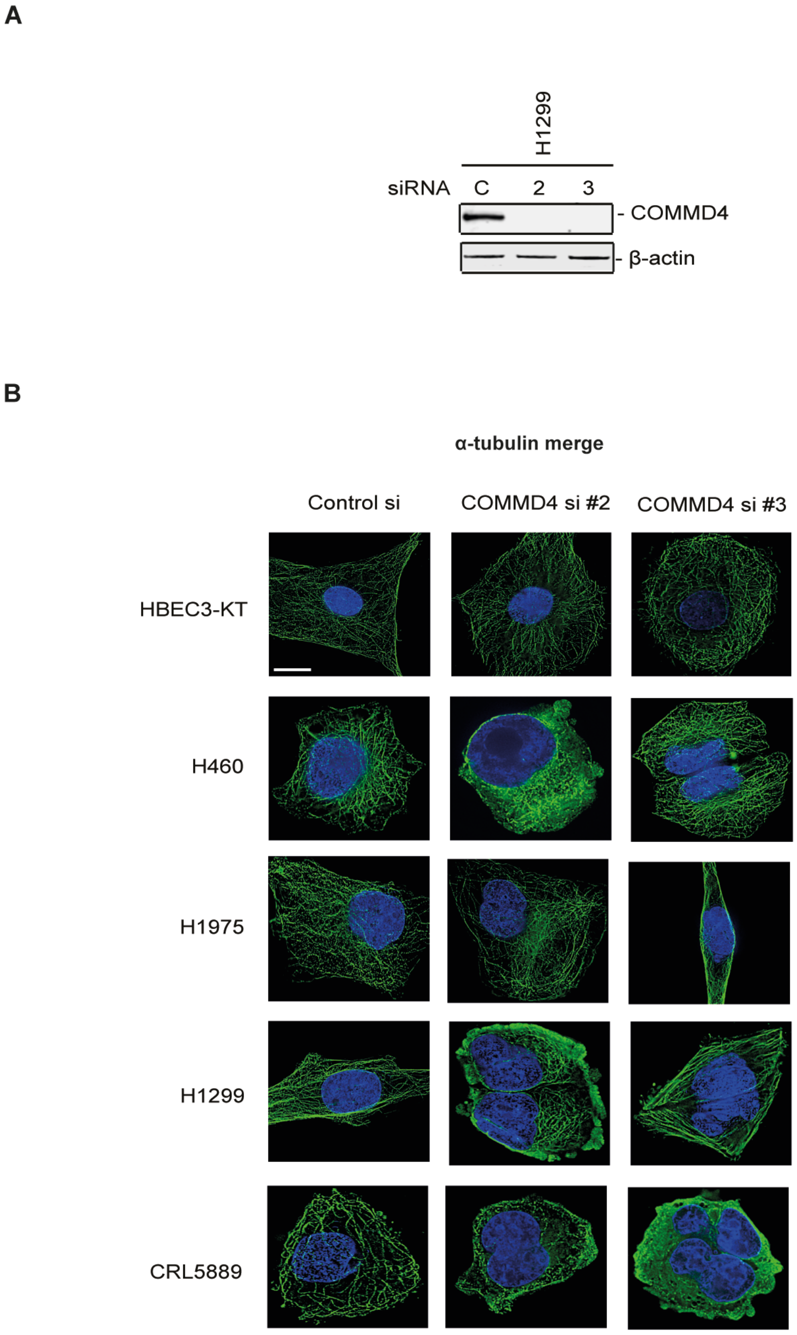

Supplementary Figure 3. COMMD4 expression and COMMD4-depletion causes mitotic catastrophe. A. An immunoblot showing the depletion of COMMD4 using control siRNA or COMMD4 siRNA #2 or #3 in the H1299 NSCLC cell line. β-actin shows the loading. B. α-tubulin staining (green) in control HBEC3-KT and H460, H1975, H1299, and CRL5889 NSCLC cell lines. DAPI shows the nucleus. Scale bar denotes 15 µm.

A

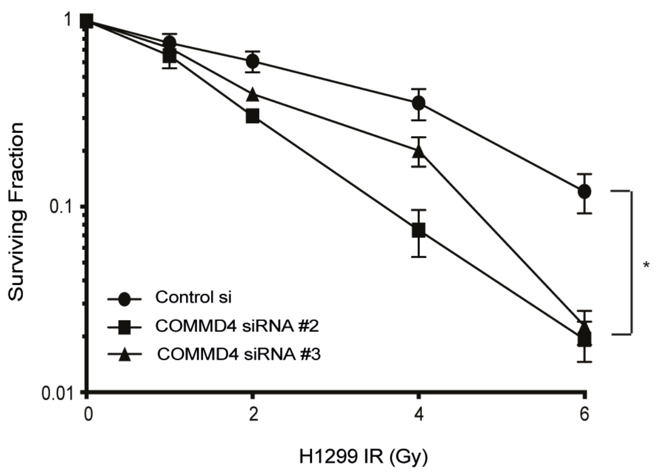

B

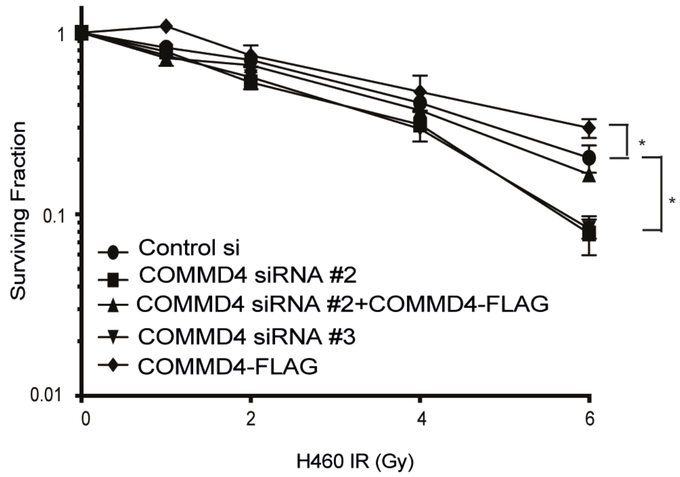

Supplementary Figure 4. Hypersensitivity to irradiation in COMMD4-depleted cells and complementation of the hypersensitivity with the overexpression of COMMD4. A. Clonogenic cell viability assay in H1299 cells transfected with control or COMMD4 siRNA (#2 or #3) and treated with varying doses of irradiation (IR). B. Clonogenic assay demonstrating the correction of the IR defect in cells depleted of COMMD4 (with siRNA #2) using a COMMD4 siRNA-resistant plasmid and overexpression of COMMD4-FLAG in the H460 cells. *; $P < 0.05$ from three independent experiments. Error bars represent mean ± SD.

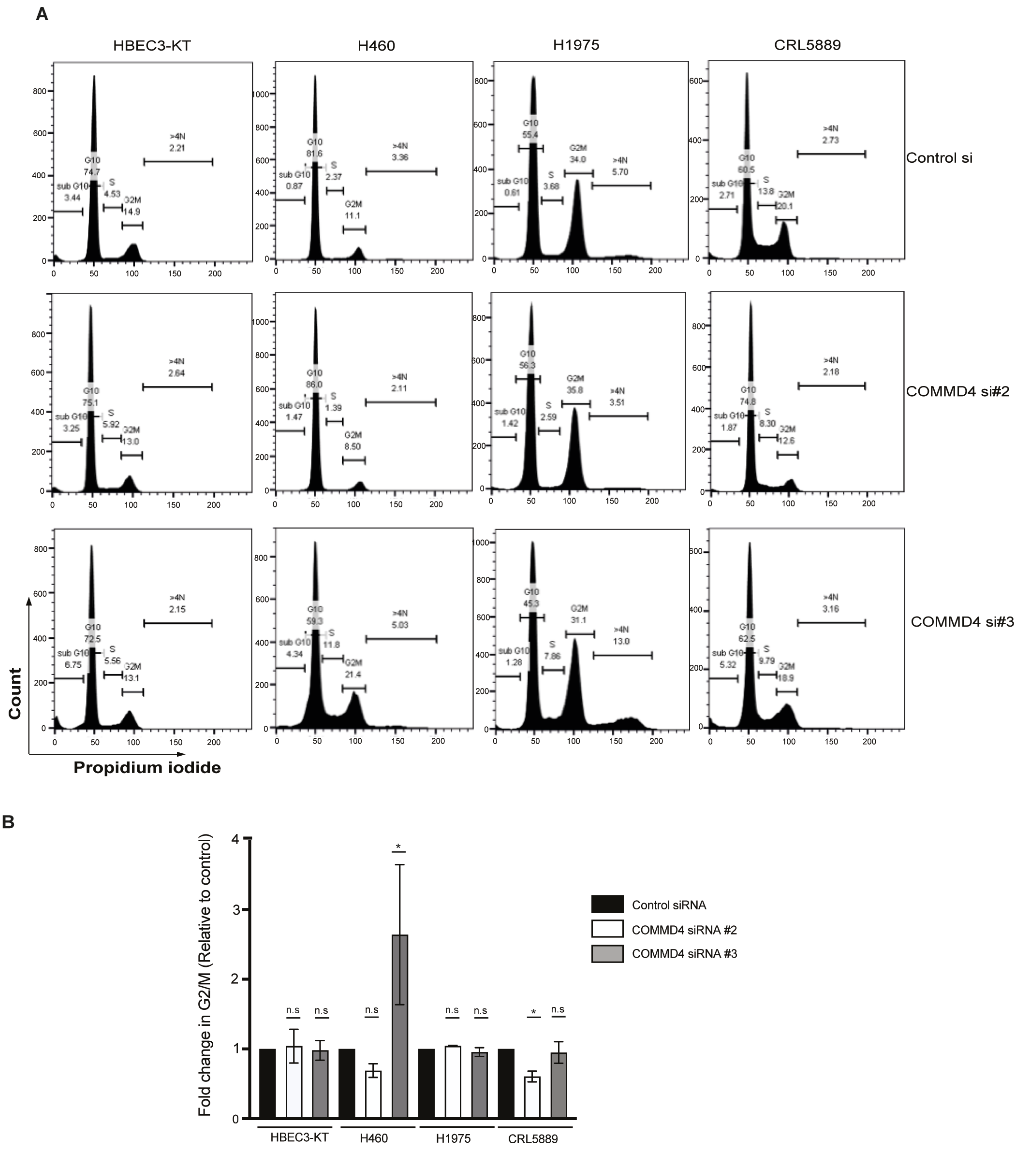

Supplementary Figure 5. Cell cycle analysis in COMMD4-depleted cells. A. Flow cytometry analyses of the DNA content profile for control siRNA and siRNA #2 and siRNA #3 treated HBEC3-KT, H460, H1975 and CRL5889 cell lines. Live cells were stained with Annexin V-488 and propidium iodide and assessed using a CytoFLEX flow cytometer. The DNA content was determined by staining the fixed cells with propidium iodide prior to flow cytometry. B. Quantification of (A) using FlowJo v10 software to quantify the proportion of cells within the G/M phase of the cell cycle in the cell lines analysed. *p < 0.05, n.s; not significant. Error bars represent mean ± S.D from three independent experiments. HBEC3-KT, control cell line and H460, H1975 and CRL5889 are NSCLC cells. sub G10; cells in sub G0/G1 phase, G10; cells in G0/G1 phase, S; cells in S phase, G2M; cells in the G2/M phase and >4N; aneuploid cells > 4N.
